# Death from Chloroform

**Published:** 1868-01

**Authors:** 


### DEATH FROM CHLOROFORM.

WE copy the following from a Minnesota paper of Feb. 5th, in reference to a coroner's inquest.

"Upon the evidence adduced, as above and heretofore presented, the Jury, after a brief consultation together, unanimously returned the following verdict:

STATE OF MINNESOTA, County of Winona, *ss.*—An inquisition taken at Winona, in the county of Winona, on the 28th day of January, A. D. 1868, before Edward Ely, Deputy Coroner of the said county of Winona, upon view of the body of Hannah Lowe, lying there dead, by the oaths of the jurors, whose names are hereunto subscribed, who being sworn to inquire on behalf of the State of Minnesota, when, how, and by what means, the said Hannah Lowe came to her death, upon their oaths do say: That she died about 4 o'clock A. M., January 23d, 1868, of congestion of the lungs, induced by the inhalation of impure chloroform, administered by Dr. T. B. Welch, of this city.

In testimony whereof, the said coroner and jurors of this inquest, have hereunto set their hands the day and year aforesaid.

E. ELY, *Deputy Coroner.*

W. J. YOUMAS, M. J. LAIRD,
CHAS. BENSON, G. W. BUSWELL,
MARK WILLSON, M. B. BUFFUM.

Thus ends the most protracted, as well as one of the most interesting examinations by a Coroner's Jury, ever held in Winona."

This adds another to the already large list of deaths, from the use of chloroform. The testimony before the jury in reference to the matter was very lengthy and complete, and by it the following facts are shown. The victim was a lady in good health, except a slight cold.

The post-mortem examination revealed the fact, that the heart and lungs, with all their connections were, previous to the administration of the chloroform, in a sound and healthy condition. The state of the lungs at the time, however, were found in a state of intense congestion, and an arrest of their circulation, to such an extent, that death occurred by asphyxia.

The lady took the chloroform at her own request, and without fear, suffered severely from the beginning, experiencing a sense of suffocating, this increasing till death, which occurred in a little over twelve hours after taking chloroform.

After recovering from the anæsthetic state, she was able with but little assistance to walk, and the operator supposed at the time she left his office that very soon all would be right, such however was not the case.

The chloroform was administered slowly and carefully, by one who had been accustomed to giving it for fourteen years, had been educated a physician and dentist, and had been more or less practicing both for fourteen years; always used the best chloroform that could be procured, in this case Powers & Whiteman's, than which none has a better reputation, and this sample was doubtless good when procured; but upon examination after this accident proved to be very impure, having mixed with it hydro-chloric acid to such an extent as to produce a peculiar appearance in the bottle, and to produce when inhaled unusual irritation and disturbance in the throat and air passages, and continued unusual symptoms.

The occasion of the adulteration does not appear in the testimony. Decomposition of the chloroform was suggested; this is hardly probable, for it appears that a very short time previous to this, chloroform from the same bottle was given to other patients, and even if a decomposition should take place—which we very much doubt, judging from the affinity of its elements—it would certainly be a very slow and gradual process. The only apparently rational conclusion is, that by some unfortunate circumstance, the chloroform had mixed with it the fatal ingredient. Various adulterations of chloroform have been suggested as rendering it dangerous, and none perhaps more so, than the one here mentioned. Great caution should therefore be exercised in selecting it, and in keeping it, while in the possession of the physician or dentist.

We know of none better than that referred to above, and as there is a difference of opinion as to whether light will decompose it or not, it is at *least* safe to keep it in the dark, and especially should great caution be exercised lest it become injured by the admixture of other substances.

The question will naturally arise in the mind here, is the operator censurable in this case? Though there may be difference of opinion as to the degree of censure that should attach to him, yet all will see that there were some things that should have received his attention that were overlooked.

The lady had taken a full meal just previous to calling at the office; the peculiar action of the chloroform upon her should have arrested his closest observation at once, and we think have stopped the administration of the agent; the action of the chloroform upon himself should also have aroused his attention; but above all, when he observed an unusual appearance of the chloroform, he should have refrained from using it.

Dr. W. says in his testimony, "I noticed an oily substance on the chloroform in the bottle, first when I commenced using it on her; supposed it to be some of the wax from the seal of the bottle, which had been dissolved; but to avoid getting the gummy substance on the handkerchief, I poured it out into another bottle; was very much affected by the chloroform while administering it to Mrs. Lowe, but supposed it was owing to my being unwell; was obliged to leave the patient and relieve myself by throwing water in my face; it never had such an effect before."

These circumstances should at once have called forth the closest scrutiny, and an absolute arrest of administration until the condition of the agent was thoroughly tested. Again, the

Dr. says, "My manner of determining the character of chloroform, is from the house where I get it; generally taste and smell of it, and judge of its effect."

Now we can readily understand how any one may place great confidence in a therapeutic agent that is universally approved for its genuineness, but we should insist that where life is at stake, the greatest caution should be exercised. The tests for chloroform are very simple and definite, and where there is a possibility of a doubt as to purity, they should be resorted to.

In this matter we would not certainly attach criminality in its odious sense to Dr. W., yet we think he can hardly hold himself free from censure; but he will have a large share of sympathy from his professional brethren. T.

